# Effect of Different Tannin Sources on Nutrient Intake, Digestibility, Performance, Nitrogen Utilization, and Blood Parameters in Dairy Cows

**DOI:** 10.3390/ani9080507

**Published:** 2019-07-31

**Authors:** Jun Zhang, Xiaofeng Xu, Zhijun Cao, Yajing Wang, Hongjian Yang, Arash Azarfar, Shengli Li

**Affiliations:** 1 State Key Laboratory of Animal Nutrition, Beijing Engineering Technology Research Center of Raw Milk Quality and Safety Control, College of Animal Science and Technology, China Agricultural University, Beijing 100193, China; 2 College of Agriculture, Ningxia University, Yinchuan 750021, China; 3 Department of Animal Science, Faculty of Agriculture, Lorestan University, P.O. Box 465, Khorramabad 68151-44316, Iran

**Keywords:** condensed tannin, hydrolyzed tannin, nitrogen utilization, dairy cows

## Abstract

**Simple Summary:**

Improving dietary nitrogen utilization efficiency or at least changing the nitrogen excretion route in lactating dairy cows, benefits both the environment and dairy economics; and reduces competition for nitrogen resources between animal feeding and human consumption. However, the effects of tannin supplements from different sources on nitrogen use in dairy cows require further investigation. This study showed that dietary supplementation with tannins in lactating dairy cows significantly decreased the milk and blood urea nitrogen concentrations, and altered the nitrogen excretion routes from the urine to the feces, which may alleviate concerns about nitrogen excretion from dairy farms to the environment.

**Abstract:**

This study investigated the effect of tannin sources on nutrient intake, digestibility, performance, nitrogen utilization, and blood parameters in lactating dairy cows. Four multiparous lactating Holstein cows were used in a balanced 4 × 4 Latin square design, with each period lasting 28 days. Cows were randomly assigned to one of four dietary treatments: Control diet (CON, a totally mixed ration without tannin supplements), control diet supplemented with 3% bayberry condensed tannins (BCT), control diet supplemented with 3% *Acacia mangium* condensed tannins (ACT), and control diet supplemented with 3% valonia hydrolyzed tannins (VHT). Dietary treatments did not significantly affect nutrient intake, milk yield or composition, microbial protein synthesis, nitrogen utilization efficiency, or plasma concentrations of glucose, non-esterified fatty acids, *β*-hydroxybutyrate, total protein, and globulin, or the albumin-to-globulin ratio. Tannin supplements decreased the apparent total tract nutrient digestibility to varying degrees and significantly decreased the milk and blood urea nitrogen contents (*p* < 0.05). Tannin supplements altered nitrogen excretion routes in lactating dairy cows, and BCT significantly decreased the urinary nitrogen excretion (*p* = 0.04). Compared with the CON, ACT, and VHT diets, BCT yielded the highest nitrogen retention and nitrogen retention-to-digestible nitrogen ratio despite having a similar nitrogen utilization efficiency (*p* < 0.05). Bayberry condensed tannin supplementation may be a potential way to improve nitrogen utilization and reduce concerns regarding nitrogen excretion in dairy cows.

## 1. Introduction

Fast growth of the global population will lead to largely increased demands for nitrogen resources and a healthy environment. Because of its low nitrogen utilization efficiency, the dairy industry has been blamed for its higher nitrogen excretion, which may detrimentally affect the water, land, air quality, ecosystem biodiversity, and human health [1]. Excessive dietary nitrogen is excreted via urine and feces, and urinary nitrogen excretion can result in increased environmental losses through nitrate leaching, NH_3_ volatilization, and nitrous oxide emissions [2,3]. In addition, dietary nitrogen resources, commonly calculated as protein and mainly supplied from soybeans, are expensive components of dairy cattle diets [1,4]. Thus, improving the dietary nitrogen utilization efficiency or at least changing the nitrogen excretion route will benefit both the environment and dairy economics as well as reduce competition for nitrogen resources between animal feeding and human consumption [1].

In recent years, plant extracts as growth promoters and natural alternatives to antibiotics have attracted the attention of ruminant nutritionists. Tannins are compounds that can improve nitrogen utilization and feed conversion efficiency, change milk fatty acid profiles, and reduce methane emission in ruminants [5,6,7]. However, studies on the effects of plant-derived tannins in ruminant nutrition are conflicting. Bhatta et al. [8] found that a low concentration (7.5%) of tamarind seed husk tannins improved the daily weight gain and milk protein content of crossbred lactating cows. Dschaak et al. [2] reported that supplementing 3% quebracho condensed tannins decreased dry matter intake (DMI), but did not affect milk production, milk composition, or N utilization efficiency for milk production. Broderick et al. [9] showed that feeding birdsfoot trefoil condensed tannins reduced the concentrations of true protein and urea nitrogen in milk, and depressed the apparent nutrient digestibility. Such discrepancies can be attributed to the tannin source, type, and dietary inclusion level [10,11,12].

Based on their structure and reactivity, tannins are categorized as either hydrolyzable or condensed [13,14]. Condensed tannins (CTs) can bind with dietary proteins and decrease their degradability in the rumen [6,12,14]. Hydrolyzable tannins (HTs) have a smaller molecular weight (500 to 3000 Da) to that of CTs (1900 to 28,000 Da), and are more easily absorbed from the intestine, thus increasing their potential toxicity to animals [6,14]. Some studies shown no difference among tannin sources on ruminal protein degradation, total tract digestibility of dietary proteins, or animal performance [15,16]. Liu et al. [17] found no toxicity to sheep supplied with 3% HTs. Most previous studies used only one type of tannin, and few studies have compared the effects of HTs and CTs on production performance in lactating dairy cows. Therefore, this study aimed to compare the effects of different tannin sources on nutrient intake, digestibility, performance, nitrogen utilization, and blood parameters in lactating dairy cows.

## 2. Materials and Methods

### 2.1. Cows, Experimental Design, and Treatments

Four lactating Holstein cows (second parity; 550 ± 30 kg of live weight; 78 ± 15 days in milk; body condition score 3.00 ± 0.25) were used in a 4 × 4 Latin square design balanced for carryover effects. Each experimental period consisted of 25 days for adaptation and 3 days for sample collection. Cows were fed a total mixed ration (TMR) ad libitum (targeting approximately 10% orts on an as-fed basis; Table 1) either supplemented with no dietary tannins (control group, CON) or supplemented with 3% bayberry condensed tannins (BCT; contained 68% purified tannins), *Acacia mangium* condensed tannins (ACT; contained 75% purified tannins), or valonia hydrolyzed tannins (VHT; contained 68% purified tannins). Those tanniniferous products (Guangxi Wuming Tannin Extract Factory, Guangxi, China) were mixed daily with the basal diet to form a premix (10% tannin and 90% basal diet, dry matter (DM) basis), and fed as a top dress. Diets were formulated to meet or exceed the nutrient requirements for dairy cows under the previously mentioned conditions [18] and were offered twice daily at 06:30 and 18:30. Cows were housed in individual tie stalls with free access to water during the experiment, and milked twice daily before feeding at 06:00 and 18:00. Animal care was in accordance with the Instructive Notions with Respect to Caring for Experimental Animals, Ministry of Science and Technology of China (13 September 2006).

### 2.2. Sampling, Data Collection, and Chemical Analyses

Feed offered and refused was recorded daily, and daily samples were collected to determine dry matter intake (DMI) and nutrient intake. Samples of ingredients, total mixed ration, and orts were dried in a forced-air oven at 65 °C for 48 h to determine DM, crude protein (CP), Ca, and P, as previously reported [19,20]. Neutral detergent fiber (NDF) and acid detergent fiber (ADF) were determined according to the previously described method [21] with heat-stable *α*-amylase and sodium sulfite, and expressed including residual ash.

Daily milk yield of the cows was recorded throughout the experiment. Milk was sampled during the morning and evening milkings on the last three consecutive days (26, 27, and 28) of each period, and pooled for each cow and period to obtain one composited milk sample per cow per period. One aliquot of milk sample was preserved with potassium dichromate, and stored at 4 °C until analyzed for protein, fat, and lactose, as described previously [22]. Another milk sample was used to determine milk urea nitrogen (MUN), as described elsewhere [23].

Total fecal and urine samples were collected on the last 3 days of each period (26, 27, and 28) as described previously [22,24] to estimate apparent digestibility parameters and N balance. Briefly, fecal collection containers were used to collect fecal samples from individual cows. The urinary collection apparatus was composed of an anterior urethra, a urine cup, and a collection barrel. The fecal and urinary amounts were recorded daily. Fecal samples for every cow were pooled per day, subsampled (approximately 400 g), and preserved by adding 1/4 weight of 10% tartaric acid. Nutrients (DM, NDF, ADF, and CP) in the feces were determined as previously described for the TMR. After filtering through four-layer gauze, the urine was stored at −20 °C for further analyses. Urinary purine derivatives (PD; uric acid and allantoin) were determined per the procedure of Chen and Gomes [25], and used to indirectly estimate the microbial protein synthesis (MPS) yield in the rumen [26]. Total nitrogen content in the feces and urine were measured using the Kjeldahl method [20]. Nitrogen retention was calculated by subtracting the N excretion in the milk, feces, and urine from the N intake [27]. Digested nitrogen was calculated as nitrogen digestibility × nitrogen intake.

Blood samples were collected from the caudal vena cava using a disposable vacuum blood collection tube containing heparin on the last day of each period. All tubes were centrifuged at 3000× *g* for 15 min to obtain plasma and stored at −20 °C until further analyses. Plasma albumin, globulin, glucose (GLU), urea nitrogen (PUN), non-esterified fatty acid (NEFA), and *β*-hydroxybutyrate (BHBA) concentrations were measured using a Hitachi automatic biochemical analyzer (Hitachi 7020; Hitachi Limited, Tokyo, Japan) with the kits supplied by the instrumentation laboratory as per our previous studies [19,22].

### 2.3. Statistical Analysis

Data on nutrient intake and digestibility, performance, nitrogen emissions, and blood parameters were analyzed using the MIXED procedure in SAS software (SAS Institute Inc., Cary, NC, USA). The model was:

Y_ijkl_ = μ + P_i_ + C_j_ + T_k_ + e_ijkl_ (1)

where Y_ijkl_ is the dependent variable under examination, μ is the overall mean, P_i_ is the fixed effect of the period (i = 4), C_j_ is the random effect of the cow (j = 4), T_k_ is the fixed effect of the dietary treatments (k = 4), and e_ijkl_ is the random error. Tukey’s test was used for multiple comparisons. The least square means were reported in the results. Significance was declared at *p* ≤ 0.05, and trends were considered as 0.05 < *p* ≤ 0.10.

## 3. Results

### 3.1. Nutrient Intake and Digestibility

The tanniniferous sources did not significantly affect the DM, NDF, ADF, or CP intakes (Table 2). The apparent total tract digestibilities of DM, NDF, ADF, and CP were lower in the ACT group compared with the other groups (*p* < 0.05; Table 3) but were similar among the other groups except for the apparent total tract digestibility of CP, which was lower in the VHT group than in the control group (*p* < 0.05; Table 3).

### 3.2. Animal Performance

Compared with the CON group, tannin treatments, regardless of their origins, significantly reduced the MUN concentration (Table 4), but did not affect milk production, fat-corrected milk (FCM), energy-corrected milk (ECM), or the milk fat, protein, and lactose percentages.

### 3.3. Nitrogen Utilization

The ACT and VHT groups had higher (*p* < 0.05) fecal excreted nitrogen amounts and fecal nitrogen excretion-to-urinary nitrogen excretion ratios than did the CON group, but a lower nitrogen retention and nitrogen retention-to-digested nitrogen ratio (NR:ND) than did the CON and BCT groups (Table 5). Among the groups, BCT excreted the least urinary nitrogen compared with the CON and VHT groups (*p* < 0.05), but the highest (*p* < 0.05) nitrogen retention and NR:ND of the three groups. MPS and nitrogen utilization efficiency were similar among the dietary treatments.

### 3.4. Blood Metabolites

Compared with CON cows, feeding cows with diets containing tanniniferous supplements significantly reduced the PUN concentration (Table 6). The BCT and VHT groups had the lowest PUN concentrations. The VHT cows had a lower (*p* < 0.05) plasma concentration of albumin than did the ACT cows. Tanniniferous supplements did not significantly affect the other plasma parameters.

## 4. Discussion

### 4.1. Nutrient Intake and Digestibility

Tannins have previously been considered antinutritional compounds because of their negative effect on DMI. However, the effects of tannins on nutrient intake have been inconsistent. Some experiments showed that 5.5–7.5% tannins inhibited the DMI in ruminants [29,30], which might be attributed to the reduced feed palatability [31] and astringency of the tannins [32]. Other studies found that dietary inclusion of tannins at levels of less than 3% of the DM either slightly increased or did not affect the DMI in ruminants [33,34,35]. Similarly, our study found that tannins did not significantly affect nutrient intake when tannin supplements at 3% of the DM were added to the diets of lactating dairy cows. Since tannins have complex compositions and structures, their sources should be considered when choosing the practical levels to add to the diets [13].

Dietary supplementation with *Acacia mangium* condensed tannins (ACT group) significantly decreased nutrient digestibility compared with that of the CON group. The high binding capacity for dietary protein and reduction of ruminal protein degradation are the most significant and well-known effects of tannins in ruminants [6,14,36]. In accordance with our results, other studies have reported that tannins can form complexes with digestive enzymes secreted by bacteria, thereby affecting their activities, and that tannins can decrease the population of some microorganisms, further reducing rumen degradability of the feed [13,37,38]. Purified tannins can decrease the populations of *Ruminococcus flavefaciens* and *Fibrobacter succinogenes* in vitro, which may adversely affect fiber degradation in the rumen [39]. Compared with the CON group, VHT only negatively affected CP digestibility, and BCT did not adversely affect nutrient digestibility. The lack of an effect of VHT on DM, NDF, or ADF digestibility may be because these parameters are more susceptible to enzymatic and nonenzymatic hydrolysis [6,40]. Hagerman et al. [41] showed that HTs did not affect nutrient digestibility in ruminants. Deaville et al. [42] found that HTs do not bind with dietary fiber, which may explain why adding valonia hydrolyzed tannins to the diet of lactating dairy cows in this study did not reduce NDF or ADF digestibility.

Reduced protein degradation in the rumen occurs when tannin-protein complexes form in the rumen and inhibit proteolytic bacterial growth and activities [2,9,38]. Tannins may also negatively affect post-ruminal protein digestibility, which may be attributed to partial dissociation of the tannin-protein complexes, formation of tannin-digestive enzyme complexes, post-ruminal formation of tannin-dietary protein complexes, and changes in intestinal protein absorption when tannins interact with the intestinal mucosa [12,36]. Some researchers have reported that CTs increase the flow and absorption of non-ammonia nitrogen (NAN) to the small intestine [13,43]; however, the exact mechanisms by which CTs alter protein digestion remain unclear. Therefore, future studies are needed to investigate the effects of different tannin sources on ruminal and post-ruminal protein digestion.

### 4.2. Animal Performance and Nitrogen Efficiency

Consistent with our results, a previous study found that tannin supplementation did not affect milk yield, 4% fat-corrected milk, energy-corrected milk, or fat, protein, and lactose contents [2]. Bhatta et al. [8] found that feeding tamarind seed husk as a tannin source at 7.5% of the diet only decreased milk protein yields in mid-lactation crossbred dairy cows.

Milk urea N has been used as a management tool to improve dairy herd nutrition and monitor the nutritional statuses of lactating dairy cows [2,44,45]. Elevated MUN indicates that lactating cows have received more protein than needed for their production levels in the diets [46,47]. Urinary N excretion has a positive linear relationship with MUN [46]. In our study, tannin-protein complex formation might have decreased protein degradation in the rumen, resulting in a decreased MUN concentration. However, tannin supplementation did not affect milk protein concentration or yield despite the decreased MUN in this study, which may have been because milk protein synthesis is a long-term and complex process, and changes in the body protein reserves are also involved [48]. In the present study, total milk protein ranged from 2.95–3.20%, and MUN concentration ranged from 9.5–14.2 mg/dL, indicating that dietary rumen degradable protein and net energy for lactation were likely balanced [45,46]. The MUN and milk protein concentrations in our study were within the target value range for lactating dairy cows [42], and tannin supplementation clearly decreased the MUN concentration. The lower MUN from the tannin supplementation coincided with the lower urinary N excretion in our study, especially in the BCT and ACT groups, which was consistent with a previous report [46].

Although feeding cows ACT and VHT led to a lower apparent total tract digestibility of CP, these supplements did not affect the MPS compared with that of the CON group, indicating that tannins will not always decrease MPS. Consistent with our study, other studies have reported that tannin supplements do not negatively affect MPS. One study found that feeding sheep *Leucaena hybrid* KX2 CT (11.6% of DM) did not significantly affect the estimated microbial protein outflow from the rumen [49]. Similarly, total purines did not markedly increase in the presence of tannins compared with the control group in vitro [16]. The effect of any dietary supplements, including tannin supplements, on MPS can be influenced by both ruminal degradability of the dietary protein and the synchrony of the nitrogen and energy release in the rumen [50].

In our study, tannin supplements did not affect the nitrogen utilization efficiency, but BCT decreased the nitrogen excretion and increased the nitrogen retention in lactating dairy cows. Consistent with previous studies [9,51], tannin supplements altered the nitrogen excretion. The increased total nitrogen excretion in ACT and VHT may be due to the decreased apparent total tract digestibility of the CP. Dschaak et al. [2] also reported that supplementation of quebracho condensed tannin extract in lactating dairy cows diets changed the N excretion route, led to less urinary N excretion but more fecal N excretion, and did not affect the N utilization efficiency for milk production. Shifting the nitrogen excretion route from the urine to the feces and forming tannin-protein complexes benefit the environment. Firstly, fecal nitrogen is primarily in the organic form, which is less volatile, whereas urinary nitrogen is largely in the form of urea, which is rapidly hydrolyzed to ammonia and nitrified to nitrate [52]. Nitrate can leach into groundwater, causing water pollution and can be converted to nitrous oxide (a greenhouse gas), accounting for approximately 65% of global anthropogenic nitrous oxide emissions [12]. Secondly, tannin-protein complexes in the feces dissociate slowly in the soil, because mineralization of the complex is inhibited, and these feces decompose more slowly than do feces without CTs [53]. Therefore, decreased urinary nitrogen excretion could reduce ammonia and nitrous oxide emissions into the atmosphere [54]. Excreted nitrogen via manure is more environmentally friendly than that excreted via urine [55]. Even though both BCT and ACT are condensed tannins, they may have different structures and biological activities, which may exert different effects on nutrient digestibility and nitrogen efficiency. Meanwhile, further work to compare the structure, biological activity, and effects on nutrient digestibility and animal performance among the same types of tannins with different sources were also warranted.

### 4.3. Blood Metabolites

Blood urea nitrogen can also be used to monitor nitrogen metabolism in ruminants, and higher blood urea nitrogen can indicate higher ruminal protein degradation [47]. Similar to the MUN, the lower PUN in tannin-supplemented cows indicates a lower protein degradation in the rumen. Blood concentrations of albumin and globulin have been considered biomarkers for inflammation in dairy cows [56,57]. Although the VHT group had a lower albumin concentration than did the ACT group, the albumin and globulin concentrations in all groups were in the normal range reported for dairy cows [58]. Because some studies have indicated that HTs and/or their intermediary metabolites may be toxic to the liver [6,36,38], caution should be used when more HTs are added to diets.

### 4.4. Limitations of This Study

A limitation of this study was a failure to measure the total polyphenolic and tannins contents after mixing the products with the rest of ingredients, which could have allowed for further inter-study comparisons. However, considering the fact that the tannin content of the basal diet was negligible, the tannin content in the experimental diets can be estimated according to the amount of tanniniferous products added to the diets. Moreover, determination of fecal endogenous nitrogen would have been helpful for explaining the negative nitrogen retention value in ACT group and nearly zero value in VHT group.

## 5. Conclusions

Compared with the control group, tannin supplements had a slight effect on nutrient intake, milk production, and milk composition. The nitrogen utilization efficiency for milk production was similar among the groups, but interestingly, tannin supplements decreased the MUN and PUN concentrations and changed the nitrogen excretion route from the urine to the feces. In addition, feeding lactating dairy cows with a diet supplemented with 3% bayberry condensed tannins decreased the total, fecal, and urinary nitrogen excretions, and increased the nitrogen retention. Our results indicate that dietary supplementation of dairy cows with 3% bayberry condensed tannins may be a potential method of producing more environmentally friendly excreta on dairy farms by changing the N excretion route from the urine to the feces, and thus alleviating public concerns about N pollution. Further studies on the effects of tannins on rumen and post-rumen metabolism in lactating dairy cows are warranted.

## Figures and Tables

**Table 1:** Ingredients and chemical compositions of the experimental diets.

Item ^1^	% DM
Ingredients	
Alfalfa hay	22.0
Chinese wildrye	33.0
Corn	28.0
Soybean meal	10.0
Wheat bran	1.5
Whole cottonseed	2.6
Premix ^2^	0.5
Calcium hydrophosphate	0.4
Limestone	0.7
Sodium bicarbonate	0.6
Magnesium oxide	0.2
Sodium chloride	0.5
Chemical compositions	
NDF	38.6
ADF	22.5
CP	15.6
NE_L_ (MJ/Kg)	6.1
Ca	0.8
P	0.4

^1^ DM, dry matter; NDF, neutral detergent fiber; ADF, acid detergent fiber; CP, crude protein; NE_L_; net energy for lactation; calculated based on National Research Council [18]. ^2^ Contained (per kg of premix; DM basis): 1,000,000 IU of vitamin A, 280,000 IU of vitamin D_3_, 10,000 IU of vitamin E, 1000 mg of vitamin PP, 3250 mg Cu, 4800 mg Mn, 12,850 mg Zn, 140 mg I, 150 mg Se, and 110 mg Co.

**Table 2:** Effect of tannin treatments on nutrient intake in lactating cows ^1^.

Item (kg/d)	Treatment				SEM	*p*-Value
	CON	BCT	ACT	VHT		
DM	19.48	20.92	19.45	19.39	2.77	0.33
NDF	7.52	8.21	7.51	7.40	0.33	0.17
ADF	4.37	4.76	4.36	4.31	0.23	0.25
CP	3.05	3.12	3.04	3.03	0.44	0.94

^1^ CON, control diet; BCT, control diet supplemented with 3% bayberry condensed tannins; ACT, control diet supplemented with 3% *Acacia mangium* condensed tannins; VHT, control diet supplemented with 3% valonia hydrolyzed tannins; SEM, standard error of the mean; DM, dry matter; NDF, neutral detergent fiber; ADF, acid detergent fiber; CP, crude protein.

**Table 3:** Effect of tannin treatments on apparent total tract digestibility of nutrients in lactating cows ^1^.

Item (%)	Treatment				SEM	*p*-Value
	CON	BCT	ACT	VHT		
DM	69.76 ^a^	68.96 ^a^	61.71 ^b^	64.96 ^ab^	3.61	0.03
NDF	58.54 ^a^	55.53 ^ab^	50.96 ^b^	54.16 ^ab^	1.38	0.02
ADF	53.17 ^a^	52.17 ^a^	47.56 ^b^	51.32 ^a^	1.83	0.03
CP	69.78 ^a^	71.82 ^a^	55.86 ^c^	61.47 ^b^	1.42	<0.01

^1^ CON, control diet; BCT, control diet supplemented with 3% bayberry condensed tannins; ACT, control diet supplemented with 3% *Acacia mangium* condensed tannins; VHT, control diet supplemented with 3% valonia hydrolyzed tannins; SEM, standard error of the mean; DM, dry matter; NDF, neutral detergent fiber; ADF, acid detergent fiber; CP, crude protein. ^a–d^ Means within a row with different superscripts differ significantly (*p* < 0.05).

**Table 4:** Effect of tannin treatments on performance of lactating cows ^1^.

Items	Treatment				SEM	*p*-Value
	CON	BCT	ACT	VHT		
Production, kg/d						
Milk	22.88	23.43	23.62	23.47	1.06	0.97
FCM ^2^	20.34	21.29	21.24	21.43	0.55	0.14
ECM ^3^	22.33	23.19	23.23	23.16	1.12	0.93
Compositions, %						
Fat	3.26	3.39	3.33	3.42	0.26	0.94
Protein	3.15	3.11	3.13	3.01	0.10	0.73
Lactose	4.79	4.81	4.80	4.74	0.09	0.89
MUN, mg/dL	12.42 ^a^	10.36 ^b^	10.64 ^b^	10.17 ^b^	0.78	<0.01

^1^ CON, control diet; BCT, control diet supplemented with 3% bayberry condensed tannins; ACT, control diet supplemented with 3% *Acacia mangium* condensed tannins; VHT, control diet supplemented with 3% valonia hydrolyzed tannins; SEM, standard error of the mean; FCM, 4% fat-corrected milk; ECM, energy-corrected milk; MUN, milk urea nitrogen. ^2^ FCM = 0.4 × milk yield (kg/d) + 15 × fat yield (kg/d) [26]. ^3^ ECM = 0.327 × Milk yield + 12.95 × milk fat yield + 7.2 × milk protein yield [26]. ^a–d^ Means within a row with different superscripts differ significantly (*p* < 0.05).

**Table 5:** Effect of tannin treatments on ruminal microbial protein synthesis and nitrogen utilization in lactating cows ^1^.

Items	Treatment				SEM	*p*-Value
	CON	BCT	ACT	VHT		
Intake of nitrogen, g/d	488.0	499.2	486.4	484.8	2.75	0.96
Nitrogen digestibility, %	69.31 ^a^	71.82 ^a^	55.17 ^c^	61.14 ^b^	1.42	<0.01
MPS, mg/d	1114.8	1057.2	916.4	1041.8	127.68	0.92
Milk nitrogen yield, g/d	103.8	103.2	106.1	105.3	2.98	0.13
Fecal nitrogen excretion (FN), g/d	149.2 ^c^	140.4 ^bc^	213.3 ^a^	188.6 ^ab^	21.85	<0.01
Urinary nitrogen excretion (UN), g/d	198.1 ^a^	153.6 ^b^	174.2 ^ab^	189.3 ^a^	16.55	0.04
FN:UN	0.85 ^b^	0.91 ^ab^	1.07 ^a^	0.99 ^a^	0.04	0.04
Total nitrogen excretion in feces and urine, g/d	347.3 ^ab^	294.0 ^b^	387.5 ^a^	377.9 ^a^	23.80	<0.01
Nitrogen retention, g/d	36.9 ^b^	64.8 ^a^	−7.8 ^c^	1.6 ^d^	0.68	<0.01
NR:ND	10.9 ^b^	18.1 ^a^	−2.9 ^c^	0.51 ^d^	0.08	<0.01
Nitrogen utilization efficiency ^2^	0.23	0.23	0.24	0.23	0.01	0.99

^1^ CON, control diet; BCT, control diet supplemented with 3% bayberry condensed tannins; ACT, control diet supplemented with 3% *Acacia mangium* condensed tannins; VHT, control diet supplemented with 3% valonia hydrolyzed tannins; SEM, standard error of the mean; MPS, ruminal microbial protein synthesis; FN:UN, fecal nitrogen excretion/urinary nitrogen excretion; ND, digested nitrogen (g/d); NR:ND, nitrogen retention/digestible nitrogen. ^2^ Nitrogen utilization efficiency = (milk protein yield (kg/d) ÷ 6.38) / (crude protein intake (kg/d) ÷ 6.25) [28]. ^a–d^ Means within a row with different superscripts differ significantly (*p* < 0.05).

**Table 6:** Effect of condensed and hydrolyzable tannin additions on plasma metabolites in lactating cows ^1^.

Items	Treatment				SEM	*p*-Value
	CON	BCT	ACT	VHT		
Glucose, mmol/L	3.58	3.64	3.43	3.69	0.07	0.11
NEFA, uEq/L	91.50	105.75	104.00	109.88	8.66	0.51
BHBA, mmol/L	0.99	0.93	1.01	0.92	0.07	0.77
PUN, mmol/L	4.9 ^a^	4.3 ^b^	4.5 ^ab^	4.2 ^b^	0.09	0.02
Total protein, g/L	83.11	81.63	81.98	78.85	1.90	0.48
Albumin, g/L	36.04 ^ab^	35.79 ^ab^	36.65 ^a^	34.53 ^b^	0.42	0.03
Globulin, g/L	47.08	45.84	45.33	44.33	1.83	0.76
A:G	0.78	0.79	0.82	0.79	0.03	0.86

^1^ CON, control diet; BCT, control diet supplemented with 3% bayberry condensed tannins; ACT, control diet supplemented with 3% *Acacia mangium* condensed tannins; VHT, control diet supplemented with 3% valonia hydrolyzed tannins; SEM, standard error of the mean; NEFA, non-esterified fatty acids; BHBA, *β*-hydroxybutyrate; PUN, plasma urea nitrogen; A:G, albumin and globulin ratio. ^a–d^ Means within a row with different superscripts differ significantly (*p* < 0.05).

## References

[1] Arriola Apelo S.I., Knapp J.R., Hanigan M.D. (2014). Invited review: Current representation and future trends of predicting amino acid utilization in the lactating dairy cow. J. Dairy Sci..

[2] Dschaak C.M., Williams C.M., Holt M.S., Eun J.S., Young A.J., Min B.R. (2011). Effects of supplementing condensed tannin extract on intake, digestion, ruminal fermentation, and milk production of lactating dairy cows. J. Dairy Sci..

[3] Place S.E., Mitloehner F.M. (2010). Invited review: Contemporary environmental issues: A review of the dairy industry’s role in climate change and air quality and the potential of mitigation through improved production efficiency. J. Dairy Sci..

[4] Sinclair K.D., Garnsworthy P.C., Mann G.E., Sinclair L.A. (2014). Reducing dietary protein in dairy cow diets: Implications for nitrogen utilization, milk production, welfare and fertility. Animal.

[5] Benchaar C., McAllister T.A., Chouinard P.Y. (2008). Digestion, ruminal fermentation, ciliate protozoal populations, and milk production from dairy cows fed cinnamaldehyde, quebracho condensed tannin, or Yucca schidigera saponin extracts. J. Dairy Sci..

[6] Teferedegne B. (2007). New perspectives on the use of tropical plants to improve ruminant nutrition. Proc. Nutr. Soc..

[7] Henke A., Westreicher-Kristen E., Molkentin J., Dickhoefer U., Knappstein K., Hasler M., Susenbeth A. (2017). Effect of dietary quebracho tannin extract on milk fatty acid composition in cows. J. Dairy Sci..

[8] Bhatta R., Krishnamoorthy U., Mohammed F. (2000). Effect of feeding tamarind (*Tamarindus indica*) seed husk as a source of tannin on dry matter intake, digestibility of nutrients and production performance of crossbred dairy cows in mid-lactation. Anim. Feed Sci. Technol..

[9] Broderick G.A., Grabber J.H., Muck R.E., Hymes-Fecht U.C. (2017). Replacing alfalfa silage with tannin-containing birdsfoot trefoil silage in total mixed rations for lactating dairy cows. J. Dairy Sci..

[10] Patra A.K., Kamra D.N., Agarwal N. (2006). Effect of plant extracts on in vitro methanogenesis, enzyme activities and fermentation of feed in rumen liquor of buffalo. Anim. Feed Sci. Technol..

[11] Waghorn G.C., McNabb W.C. (2008). Consequences of plant phenolic compounds for productivity and health of ruminants. Proc. Nutr. Soc..

[12] Patra A.K., Saxena J. (2011). Exploitation of dietary tannins to improve rumen metabolism and ruminant nutrition. J. Sci. Food Agric..

[13] McMahon L.R., McAllister T.A., Berg B.P., Majak W., Acharya S.N., Popp J.D., Coulman B.E., Wang Y., Cheng K.J. (2000). A review of the effects of forage condensed tannins on ruminal fermentation and bloat in grazing cattle. Can. J. Plant Sci..

[14] Aboagye I.A., Oba M., Castillo A.R., Koenig K.M., Iwaasa A.D., Beauchemin K.A. (2018). Effects of hydrolyzable tannin with or without condensed tannin on methane emissions, nitrogen use, and performance of beef cattle fed a high-forage diet. J. Anim. Sci..

[15] Rivera-Méndez C., Plascencia A., Torrentera N., Zinn R.A. (2016). Effect of level and source of supplemental tannin on growth performance of steers during the late finishing phase. J. Appl. Anim. Res..

[16] Getachew G., Pittroff W., Putnam D.H., Dandekar A., Goyal S., DePeters E.J. (2008). The influence of addition of gallic acid, tannic acid, or quebracho tannins to alfalfa hay on in vitro rumen fermentation and microbial protein synthesis. Anim. Feed Sci. Technol..

[17] Liu H., Vaddella V., Zhou D. (2011). Effects of chestnut tannins and coconut oil on growth performance, methane emission, ruminal fermentation, and microbial populations in sheep. J. Dairy Sci..

[18] NRC (2001). Nutrient Requirements of Dairy Cattle.

[19] Zhang J., Shi H., Wang Y., Li S., Zhang H., Cao Z., Yang K. (2018). Effects of limit-feeding diets with different forage-to-concentrate ratios on nutrient intake, rumination, ruminal fermentation, digestibility, blood parameters and growth in Holstein heifers. Anim. Sci. J..

[20] AOAC (2000). Association of Official Analytical Chemists.

[21] Van Soest P.J., Robertson J.B., Lewis B.A. (1991). Methods for Dietary Fiber, Neutral Detergent Fiber, and Nonstarch Polysaccharides in Relation to Animal Nutrition. J. Dairy Sci..

[22] Shi H.T., Li S.L., Cao Z.J., Wang Y.J., Alugongo G.M., Doane P.H. (2015). Effects of replacing wild rye, corn silage, or corn grain with CaO-treated corn stover and dried distillers grains with solubles in lactating cow diets on performance, digestibility, and profitability. J. Dairy. Sci..

[23] Ekinci C., Broderick G.A. (1997). Effect of processing high moisture ear corn on ruminal fermentation and milk yield. J. Dairy Sci..

[24] Cao Z.J., Ma M., Yan X.Y., Li S.L., Zhang X.M. (2009). A simple urine-collecting apparatus and method for cows and heifers. J. Dairy Sci..

[25] Chen X.B., Gomes M.J. (1992). Estimation of Microbial Protein Supply to Sheep and Cattle Based on Urinary Excretion of Purine Derivatives—An Overview of Technical Details.

[26] Zhou X.Q., Zhang Y.D., Zhao M., Zhang T., Zhu D., Bu D.P., Wang J.Q. (2015). Effect of dietary energy source and level on nutrient digestibility, rumen microbial protein synthesis, and milk performance in lactating dairy cows. J. Dairy Sci..

[27] Ali A.I., Wassie S.E., Korir D., Merbold L., Goopy J.P., Butterbach-Bahl K., Dickhoefer U., Schlecht E. (2019). Supplementing Tropical Cattle for Improved Nutrient Utilization and Reduced Enteric Methane Emissions. Animals.

[28] Broderick G.A., Stevenson M.J., Patton R.A. (2009). Effect of dietary protein concentration and degradability on response to rumen-protected methionine in lactating dairy cows. J. Dairy Sci..

[29] McNabb W.C., Waghorn G.C., Peters J.S., Barry T.N. (1996). The effect of condensed tannins in *Lotus pedunculatus* on the solubilization and degradation of ribulose-1, 5-bisphosphate carboxylase (EC 4.1.1.39; Rubisco) protein in the rumen and the sites of Rubisco digestion. Br. J. Nutr..

[30] Barry T.N., McNabb W.C. (1999). The implications of condensed tannins on the nutritive value of temperate forages fed to ruminants. Br. J. Nutr..

[31] Cooper S.M., Owen-Smith N. (1985). Condensed tannins deter feeding by browsing ruminants in a South African savanna. Oecologia.

[32] Landau S., Perevolotsky A., Bonfil D., Barkai D., Silanikove N. (2000). Utilization of low quality resources by small ruminants in Mediterranean agro-pastoral systems: The case of browse and aftermath cereal stubble. Livest. Prod. Sci..

[33] Carulla J.E., Kreuzer M., Machmüller A., Hess H.D. (2005). Supplementation of *Acacia mearnsii* tannins decreases methanogenesis and urinary nitrogen in forage-fed sheep. Aust. J. Agric. Res..

[34] Śliwiński B., Kreuzer M., Sutter F., Machmüller A., Wettstein H.R. (2004). Performance, body nitrogen conversion and nitrogen emission from manure of dairy cows fed diets supplemented with different plant extracts. J. Anim. Feed Sci..

[35] Liu H.W., Zhou D.W., Li K. (2013). Effects of chestnut tannins on performance and antioxidative status of transition dairy cows. J. Dairy Sci..

[36] Frutos P., Hervás G., Giráldez F.J., Mantecón A.R. (2004). Tannins and ruminant nutrition. Span. J. Agric. Res..

[37] Lee J.H., Vanguru M., Kannan G., Moore D.A., Terrill T.H., Kouakou B. (2009). Influence of dietary condensed tannins from sericea lespedeza on bacterial loads in gastrointestinal tracts of meat goats. Livest. Sci..

[38] McSweeney C.S., Palmer B., McNeill D.M., Krause D.O. (2001). Microbial interactions with tannins: Nutritional consequences for ruminants. Anim. Feed Sci. Technol..

[39] Jayanegara A., Goel G., Makkar H.P.S., Becker K. (2015). Divergence between purified hydrolysable and condensed tannin effects on methane emission, rumen fermentation and microbial population in vitro. Anim. Feed Sci. Technol..

[40] O’Donovan L., Brooker J.D. (2001). Effect of hydrolysable and condensed tannins on growth, morphology and metabolism of Streptococcus gallolyticus (*S. caprinus*) and Streptococcus bovis. Microbiology.

[41] Hagerman A.E., Robbins C.T., Weerasuriya Y., Wilson T.C., McArthur C. (1992). Tannin Chemistry in Relation to Digestion. J. Range Manag..

[42] Deaville E.R., Givens D.I., Mueller-Harvey I. (2010). Chestnut and mimosa tannin silages: Effects in sheep differ for apparent digestibility, nitrogen utilisation and losses. Anim. Feed Sci. Technol..

[43] Wang Y., Waghorn G.C., Barry T.N., Shelton I.D. (1994). The effect of condensed tannins in *Lotus corniculatus* on plasma metabolism of methionine, cystine and inorganic sulphate by sheep. Br. J. Nutr..

[44] Jonker J.S., Kohn R.A., Erdman R.A. (1999). Milk urea nitrogen target concentrations for lactating dairy cows fed according to National Research Council recommendations. J. Dairy Sci..

[45] Hof G., Vervoorn M.D., Lenaers P.J., Tamminga S. (1997). Milk Urea Nitrogen as a Tool to Monitor the Protein Nutrition of Dairy Cows. J. Dairy Sci..

[46] Jonker J.S., Kohn R.A., Erdman R.A. (1998). Using Milk Urea Nitrogen to Predict Nitrogen Excretion and Utilization Efficiency in Lactating Dairy Cows. J. Dairy Sci..

[47] Broderick G.A., Clayton M.K. (1997). A statistical evaluation of animal and nutritional factors influencing concentrations of milk urea nitrogen. J. Dairy Sci..

[48] Tedeschi L.O., Seo S., Fox D.G., Ruiz R. (2006). Accounting for energy and protein reserve changes in predicting diet-allowable milk production in cattle. J. Dairy Sci..

[49] McNeill D.M., Komolong M.K., Gobius N., Barber D., Brooker J. (2000). Influence of Dietary Condensed Tannin on Microbial Crude Protein Supply in Sheep. Tannins in Livestock and Human Nutrition.

[50] Reynolds C.K., Kristensen N.B. (2008). Nitrogen recycling through the gut and the nitrogen economy of ruminants: An asynchronous symbiosis. J. Anim. Sci..

[51] Greenwood S.L., Edwards G.R., Harrison R. (2012). Short communication: Supplementing grape marc to cows fed a pasture-based diet as a method to alter nitrogen partitioning and excretion. J. Dairy Sci..

[52] Eckard R.J., Grainger C., De Klein C. (2010). Options for the abatement of methane and nitrous oxide from ruminant production: A review. Livest. Sci..

[53] Powell J.M., Broderick G.A., Grabber J.H., Hymes-Fecht U.C. (2009). Technical note: Effects of forage protein-binding polyphenols on chemistry of dairy excreta. J. Dairy Sci..

[54] De Klein C.A.M., Eckard R.J. (2008). Targeted technologies for nitrous oxide abatement from animal agriculture. Aust. J. Exp. Agric..

[55] Mlambo V., Smith T., Owen E., Mould F.L., Sikosana J.L.N., Mueller-Harvey I. (2004). Tanniniferous *Dichrostachys cinerea* fruits do not require detoxification for goat nutrition: In sacco and in vivo evaluations. Livest. Prod. Sci..

[56] Bionaz M., Trevisi E., Calamari L., Librandi F., Ferrari A., Bertoni G. (2007). Plasma paraoxonase, health, inflammatory conditions, and liver function in transition dairy cows. J. Dairy Sci..

[57] Batistel F., Arroyo J.M., Garces C., Trevisi E., Parys C., Ballou M.A., Cardoso F.C., Loor J.J. (2018). Ethyl-cellulose rumen-protected methionine alleviates inflammation and oxidative stress and improves neutrophil function during the periparturient period and early lactation in Holstein dairy cows. J. Dairy Sci..

[58] Alberghina D., Giannetto C., Vazzana I., Ferrantelli V., Piccione G. (2011). Reference intervals for total protein concentration, serum protein fractions, and albumin/globulin ratios in clinically healthy dairy cows. J. Vet. Diagn. Investig..

